# Enlarged Thalamic Volumes and Increased Fractional Anisotropy in the Thalamic Radiations in Veterans with Suicide Behaviors

**DOI:** 10.3389/fpsyt.2013.00083

**Published:** 2013-08-12

**Authors:** Melissa Lopez-Larson, Jace B. King, Erin McGlade, Elliott Bueler, Amanda Stoeckel, Daniel J. Epstein, Deborah Yurgelun-Todd

**Affiliations:** ^1^The Brain Institute, University of Utah, Salt Lake City, UT, USA; ^2^University of Utah School of Medicine, Salt Lake City, UT, USA; ^3^George E. Whalen Department of Veterans Affairs Medical Center, VA VISN 19 Mental Illness Research, Education and Clinical Center (MIRREC), Salt Lake City, UT, USA

**Keywords:** suicide, thalamus, fractional anisotropy, MRI, veterans

## Abstract

Post-mortem studies have suggested a link between the thalamus, psychiatric disorders, and suicide. We evaluated the thalamus and anterior thalamic radiations (ATR) in a group of Veterans with and without a history of suicidal behavior (SB) to determine if thalamic abnormalities were associated with an increased risk of SB. Forty Veterans with mild traumatic brain injury (TBI) and no SB (TBI-SB), 19 Veterans with mild TBI and a history of SB (TB + SB), and 15 healthy controls (HC) underwent magnetic resonance imaging scanning including a structural and diffusion tensor imaging scan. SBs were evaluated utilizing the Columbia Suicide Rating Scale and impulsivity was measured using the Barratt Impulsiveness Scale (BIS). Differences in thalamic volumes and ATR fractional anisotropy (FA) were examined between (1) TBI + SB versus HC and (2) TBI + SB versus combined HC and TBI-SB and (3) between TBI + SB and TBI-SB. Left and right thalamic volumes were significantly increased in those with TBI + SB compared to the HC, TBI-SB, and the combined group. Veterans with TBI + SB had increased FA bilaterally compared to the HC, HC and TBI-SB group, and the TBI-SB only group. Significant positive associations were found for bilateral ATR and BIS in the TBI + SB group. Our findings of thalamic enlargement and increased FA in individuals with TBI + SB suggest that this region may be a biomarker for suicide risk. Our findings are consistent with previous evidence indicating that suicide may be associated with behavioral disinhibition and frontal-thalamic-limbic dysfunction and suggest a neurobiologic mechanism that may increase vulnerability to suicide.

## Introduction

Suicide represents a major global health problem as well as a significant personal tragedy. The return of Veterans from Operation Enduring Freedom/Operation Iraqi Freedom (OEF/OIF) has emphasized the crucial importance of understanding the social, environmental, and neurobiological factors associated with suicidal behavior (SB) in both a Veteran and civilian population. However, the identification of relevant factors has often been complicated by the presence of both state and trait factors, many of which may be related to psychiatric disorders. Over the past decade, several authors have suggested that risk for suicide may be independent of a specific psychiatric disorder and may have distinct trait neurobiological expressions (1–5). For instance, biomarkers such as decreased presynaptic serotonin transporter (SERT) sites in the ventromedial prefrontal cortex (PFC) and low cerebrospinal fluid 5-hydroxyindole acetic acid have been shown to be related to suicidality and not to a specific psychiatric disorder (6–8).

Neuroimaging studies have also found regions within the fronto-striatal-limbic circuit that may be associated with SBs, irrespective of diagnosis. For example, Monkul and colleagues reported reduced orbitofrontal cortex (OFC) and increased amygdala gray matter volumes in individuals with depression and SB compared to individuals with depression alone (9). In a recent voxel-based morphometry study, 15 depressed high-risk patients, i.e., individuals who have previously attempted suicide or have a first-degree relative who attempted suicide, 15 depressed non-high-risk for suicide patients, and 30 matched healthy controls (HC) were evaluated (3). The authors reported that participants who were at high-risk for suicide showed significantly decreased gray matter density in fronto-striatal-limbic network brain regions in contrast to HC, and in rostral anterior cingulate cortex (ACC) in contrast to non-high-risk patients who also suffered from major depressive disorder (MDD). In a follow-up study by the same group, cortical thickness in dorsolateral PFC, ventrolateral PFC, and ACC were reduced in the high-risk compared to the non-high-risk for suicide group (10). Furthermore, reduced OFC and ACC volumes have been reported in individuals with a history of suicide attempts in schizophrenic (11) and bipolar disorder (BD) (12) populations.

Early work from Ahearn et al. (13) applied structural magnetic resonance imaging (MRI) and showed that, compared to unipolar depressed patients without a history of a suicide attempt, matched unipolar depressed patients with a previous attempt showed significantly more subcortical gray matter hyperintensities and periventricular white matter hyperintensities. These findings were consistent with those from Ehrlich and colleagues who found that patients with unipolar depression and white matter hyperintensities had a higher prevalence of suicide attempts (14). Pompili and colleagues also reported that among patients with MDD and BD (BD-I, BD-II) with and without a history of suicide attempt, those with a suicide attempt history were more likely to have white matter hyperintensities. In addition, the presence of these white matter changes increased the risk for SB by eight times (15, 16). These results indicate that white matter abnormalities, reflected by the presence of hyperintensities, are seen in suicidal individuals and further suggest that abnormalities in brain white matter integrity may play a role in risk for suicide. Other morphometric MRI studies of suicide have yielded findings of larger frontal white matter volume in patients with a history of a suicide attempt, which were significantly correlated with self-aggression (17). More recently Matsuo and colleagues reported that smaller anterior corpus callosum (CC) volume is highly correlated with impulsivity in suicidal patients (18) lending further support for the presence of frontal abnormalities, particularly white matter changes in individuals with SB. Taken together, these data suggest that risk factors for suicide may have separate gray and white matter neurobiological underpinnings than those alterations associated with specific psychiatric disorders (1, 3).

Currently there exists a paucity of conclusive findings regarding the relationship between suicide risk and traumatic brain injury (TBI). Of particular concern is the influx of TBI among Veterans returning from war zones in which they are frequently exposed to blast forces frequently resulting in disability. To date, over 320,000 soldiers have reported a possible TBI from OIF/OEF, and it has been estimated that as many as 60% of returning war Veterans have sustained a TBI (19, 20). In closed-head injuries, such as those suffered by soldiers exposed to blast overpressurization waves, the head is subject to forces that commonly result in shearing straining of neuronal fibers (21). This injury pattern, broadly referred to as diffuse axonal injury (DAI), seems to exert the greatest effect on structures in and around the junction between white and gray matter and most often occurs in the frontal and temporal areas, internal capsule, thalamus, and other deep gray matter structures. Conflicting reports regarding the relationship between TBI and suicide behaviors underscore the need to better understand the neuropathology of TBI and how it may affect and be affected by the risk of suicide both pre- and post-injury.

A post-mortem study of a mixed patient population, including MDD, BD, and schizophrenia, in which half died by suicide found enlarged thalamic volumes in those individuals who had an MDD diagnosis, died by suicide, or had the short allele (ss) for a genetic variant for the SERT gene (22). Individuals with the short genotype had 11% larger total thalamic volume than controls (22). Furthermore individuals with major depression had 12% larger total thalamic volumes and individuals who had completed suicide demonstrated an 8% increase in total thalamic volume compared to controls. Authors concluded that individuals with enlarged thalami might have an anatomical vulnerability to stress deriving from alteration of thalamocortical circuitry, which may predispose individuals to symptoms of depression and to behaviors such as suicide (22). It has been shown that the SERT regulates serotonin levels in the brain by transporting serotonin from the extracellular space into the neuron. A genetic polymorphism in the promotor region of the SERT gene (5-HTTLPR) results in a short or long SERT allele. This genetic variation can alter SERT expression (23, 24) and the short SERT allele has been associated with an increased incidence of major depression and SB (25, 26). Finally, Pezawas and colleagues (27) found that normal, non-psychiatric SERT-ss carriers have a 25% reduction in the volume of the ACC and a 15% reduction in the amygdala volume. Together these findings suggest that variations in SERT affect the structure and function of fronto-thalamic-limbic systems.

The association of impulsivity, aggression, and suicide risk has been reported in the literature (17, 18, 28, 29). For example, Mann et al., reported a close association between major depression, attempted suicide, and aggression in which patients with major depression that had attempted suicide had higher overall levels of lifetime aggression and impulsivity than non-attempters (28). In addition, in a study of Veterans with mild TBI, cingulum fractional anisotropy (FA) was positively correlated with current suicidal ideation and measures of impulsivity (29). Several other neuroimaging studies have also reported volumetric and metabolic abnormalities of the PFC in aggressive, impulsive, and suicidal individuals (9, 11, 17, 30, 31). Although there do not appear to be any neuroimaging studies directly implicating the thalamus, anterior limb of the internal capsule (ALIC), or anterior thalamic radiations (ATR) in aggressive and impulsive behaviors, several studies discuss the implications of thalamocortical circuitry in which thalamic projections appear to directly affect frontal-executive functioning. For example, Mamah and colleagues (32) demonstrated a relationship between ATR fiber integrity and cognitive performance in schizophrenic patients, in which decreased anisotropy within this region correlated with decreased executive function. Coenen and colleagues used diffusion tensor imaging (DTI) fiber tracking to describe anatomical convergence of the medial forebrain bundle and ATR and suggested this convergence may sustain equilibrium between positive and negative emotions, as well as reward seeking and punishment functions evident in addictions and depression (33).

The overall aim of the proposed study was to examine the neurobiological correlates of trait related risk factors for suicide in a cohort of Veterans with TBI with and without SB, matched for depression, posttraumatic stress disorder (PTSD), and TBI severity indices. Given the recent post-mortem study of enlarged thalamic volumes in individuals who committed suicide and the paucity of data examining the morphological changes of the thalamus in individuals with SB, we performed a volumetric analysis of thalamic volumes in Veterans with TBI with and without SB. We hypothesized that individuals with SB would have enlarged thalamic volumes compared to a HC population and to individuals with TBI without SB. In addition, we examined the FA of the ATR to determine if FA would have a corresponding increase or decrease in association with hypothesized changes in thalamic volumes in our TBI with SB group. We then examined our morphological findings in relation to symptoms of impulsivity and suicide.

## Materials and Methods

### Subjects

The Institutional Review Boards at the University of Utah and the George E. Whalen Department of Veterans Affairs (VA) Medical Center approved this study. All subjects provided written informed consent prior to participation in this study. A total of 74 male subjects including, 40 Veterans with mild TBI and no SB (TBI-SB) (age = 34.6 ± 8.1 years), 19 Veterans with mild TBI and a history of SB (TB + SB) (age = 38.0 ± 7.8 years), and 15 HC (age = 36.5 ± 11.5 years) were recruited from the George E. Whalen VA Medical Center and the community via local advertisements and by word of mouth. Inclusion criteria for all participants in this analysis were: ages 18–55 years old. Demographic information on the diagnostic groups is shown in Table 1. Participants were considered to have a mild TBI if they reported an injury event to the head followed by an alteration or loss of consciousness (34). The Ohio State University-TBI Identification Method (OSU-TBI) was administered during clinical evaluation to quantify presence, number, and severity of lifetime TBI injuries (35). Mild brain injury events were defined as a loss of consciousness for 30 min or less.

**Table 1:** **Demographic and clinical measures**.

	HC (*n* = 15)		TBI-SB (*n* = 40)		TBI + SB (*n* = 19)		HC versus TBI + SB	TBI-SB versus TBI + SB	HC and TBI-SB versus TBI + SB
	Mean	SD	Mean	SD	Mean	SD	*p*-Value	*p*-Value	*p*-Value
Age	36.47	11.51	34.60	8.10	38.00	7.77	ns	ns	ns
GAF	80.57	6.97	67.08	13.83	53.42	6.19	<0.001	<0.001	<0.001
Education	14.73	2.52	14.35	1.99	13.53	0.90	0.10	0.03	0.01
Hollingshead	32.09	13.19	33.15	11.62	27.32	8.31	ns	0.06	0.03
HAM-D	2.73	3.13	9.78	7.74	15.00	7.68	<0.001	0.02	0.001
HAM-A	3.73	5.16	13.40	11.65	14.95	8.13	<0.001	ns	ns
BIS planning	25.80	5.24	25.95	4.57	28.63	4.18	0.09	0.04	0.03
BIS motor	22.73	4.53	24.13	5.15	25.47	5.89	ns	ns	ns
BIS attention	18.87	2.61	19.25	3.75	22.00	3.87	0.01	0.01	<0.01
BIS total	67.40	9.19	69.33	10.43	76.11	10.83	0.02	0.03	0.02
Vocabulary	41.27	7.97	42.88	7.39	39.79	9.36	ns	ns	ns
Age at first injury (years)	–	–	21.49	6.08	19.37	6.61	–	ns	–
Number of mild TBI	–	–	4.50	7.25	3.89	3.18	–	ns	–
Number of mild TBI with LOC	–	–	0.68	0.83	1.21	1.03	–	0.04	–
Intensity of ideation	–	–	–	–	17.00	5.26	–	–	–
Number of suicide attempts	–	–	–	–	0.95	1.03	–	–	–
Number of suicide behaviors	–	–	–	–	2.05	1.61	–	–	–

			*n*	%	*n*	%		χ^2^	

PTSD (%)	–	–	23	57.50	14	73.68	–	0.26	–
MDD (%)	–	–	23	57.50	14	73.68	–	0.26	–
MDD Current (%)	–	–	15	37.50	7	36.84	–	0.96	–
Both PTSD and MDD	–	–	16	40.00	12	63.16	–	0.10	–

Comparison HC did not have a current major DSM-IV Axis I diagnosis, including substance use disorders (SUD) other than nicotine, based on clinical interviews. Exclusion criteria for all subjects included major sensorimotor handicaps (e.g., deafness, blindness, paralysis); estimated full scale IQ < 80; history of claustrophobia, autism, schizophrenia, anorexia nervosa or bulimia, active medical or neurological disease other than TBI that would impact neurobiology or brain function; history of electroconvulsive therapy; and metal fragments or implants that would be contraindicated in an MRI.

### Assessment measures

All participants, including HC, completed the Structured Clinical Interview for DSM-IV Patient Version (SCID-I/P) (36) administered by either a board-certified psychiatrist (MLL) or a licensed clinical psychologist (ECM). Inter- and intra-rater reliability has previously been established by Dr. Lopez-Larson and Dr. Yurgelun-Todd on prior studies. However, reliability estimate for other raters, EM and AS were not performed at time of study. Therefore, all SCID evaluations and diagnoses were reviewed in collaboration with all raters (MLL, DYT, EM and AS) and consensus diagnosis/diagnoses were obtained.

Measures of current clinical status were obtained prior to scanning and included the Hamilton Depression Rating Scale (HAM-D) (37) and the Hamilton Anxiety Scale (HAM-A) (38). Social status was assessed using modifications of the Hollingshead Four-Factor Index of Socioeconomic Status (39). Full scale IQ was estimated using the Vocabulary subtest as it has been thought to be the best estimate of general intelligence and pre-morbid level of functioning (40). The DSM-IV-TR Global Assessment of Functioning (GAF) (41) was used to assess global functioning using a scale from 1 (worst) to 100 (best). Current medications for each participant were obtained via patient interview and confirmed by chart review. Medications included in the hypnotic/non-benzodiazepine group included low dose trazodone (150 mg a day or less), prazosin, zolpidem and eszopiclone.

The Columbia Suicide Severity Rating Scale (C-SSRS) was administered to all participants to assess for the presence of past and current suicidal ideation and SB (42). In addition, the C-SSRS was used to quantify the intensity of the suicidal ideation at its most severe time point in the life history of an individual and was utilized to quantify the number of SBs. SBs were defined as an actual suicide attempt, an interrupted attempt, or an aborted/self-interrupted attempt. Impulsivity was measured using the Barratt Impulsiveness Scale (BIS) (43). The BIS is a 30-item self-report questionnaire designed to assess three classes of impulsivity, including attention, motor, and non-planning categories as well as a total impulsivity score.

### Magnetic resonance imaging

Structural imaging was performed at the Utah Center for Advanced Imaging Research (UCAIR) using a 3 T Siemens Trio scanner. Structural imaging data was acquired using a T1-weighted 3D MPRAGE GRAPPA sequence acquired sagittally using a 12-channel head coil with TE/TR/TI = 3.38 ms/2.0 s/1.1 s, 8 °flip, 256 × 256 acquisition matrix, 256 mm^2^ FOV, 160 slices, 1.0 mm slice thickness. A DT-MRI GRAPPA sequence was also obtained utilizing 64 directions, two diffusion weightings: *b* = 0, 1000 s/mm^2^, TE/TR = 88 ms/9 s; matrix = 128 × 128 on 256 × 256 on FOV; 2 × 2× 2 isotropic voxels, slice thickness = 2.0 mm with 0 gap, and 70 slices. The original imaging data were transferred from the scanner in the DICOM format and coded. All MRI scans were read by a neuroradiologist to rule out gross pathology.

#### Structural MRI data

**Figure 1:**
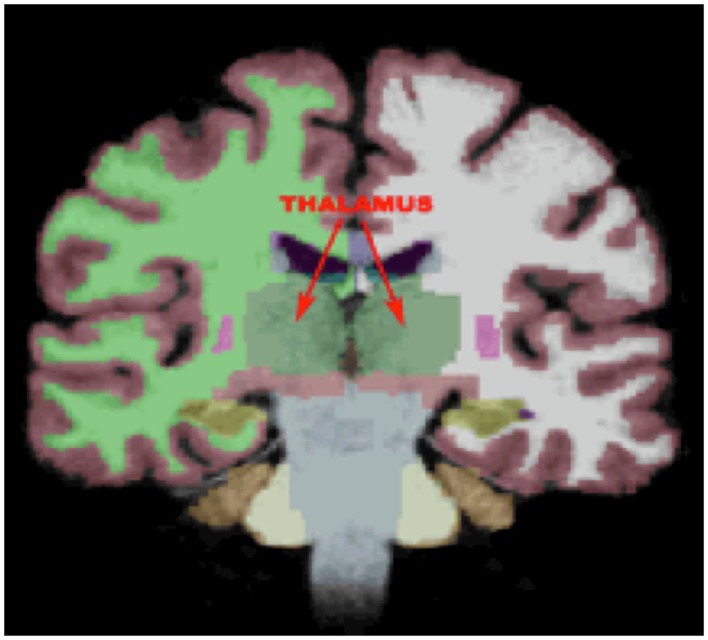
**FreeSurfer output showing left and right thalamic regions of interest**.

Thalamic volumes for each participant were calculated within the FreeSurfer image analysis environment, which is documented and freely available for download online^1^. A description of morphometric processing tailored to the needs of this study is extracted from the written description of morphometric procedures provided at the FreeSurfer website. This processing includes motion correction, removal of non-brain tissue using a hybrid watershed/surface deformation procedure (44), automated Talairach transformation, segmentation of the subcortical white matter and deep gray matter volumetric structures (45, 46), intensity normalization (47), tessellation of the gray matter white matter boundary, automated topology correction (48, 49), and surface deformation following intensity gradients to optimally place the gray/white and gray/cerebrospinal fluid borders at the location where the greatest shift in intensity defines the transition to the other tissue class (50–52). FreeSurfer morphometric procedures have been demonstrated to show good test–retest reliability across scanner manufacturers and across field strengths (53). Quality control was performed by a trained operator (JBK) throughout MRI processing within the FreeSurfer environment via manual visual inspection of each subjects’ output to ensure proper output integrity. Right and left thalamic volumetric measures were obtained from the aseg statistical output from FreeSurfer (See Figure 1).

#### Diffusion tensor imaging

The diffusion weighted imaging data was analyzed using FSL’s FDT (FMRIB’s Diffusion Toolbox, please see website for detailed methods and reference list^2^) (54–56). A description of the DTI imaging processing is extracted from the written description of the procedures provided at the FSL website. The DTI data was visually inspected for significant distortion, motion, and eddy artifact prior to processing and post-processing to ensure data integrity. Then the data was processed with tools within FDT, including eddy current and motion correction, brain extraction (57), and fitting a tensor model to the raw diffusion data. These steps resulted in extraction of FA. All subjects’ FA data were then aligned into a common space using the non-linear registration tool FNIRT (58, 59), which uses a b-spline representation of the registration warp field (60). The target image used in the registrations was chosen to be the most “typical” subject in the study in order to generate a study specific FA target image. This target image was then affine-aligned into MNI152 standard space. All participants’ images were then transformed into 1 mm × 1 mm × 1 mm MNI152 space by combining the non-linear transform to the target FA image with the affine transform from that target to MNI152 space. This results in a standard-space version of each subject’s FA image, which is next merged into a single FA 4D image containing all participants.

**Figure 2:**
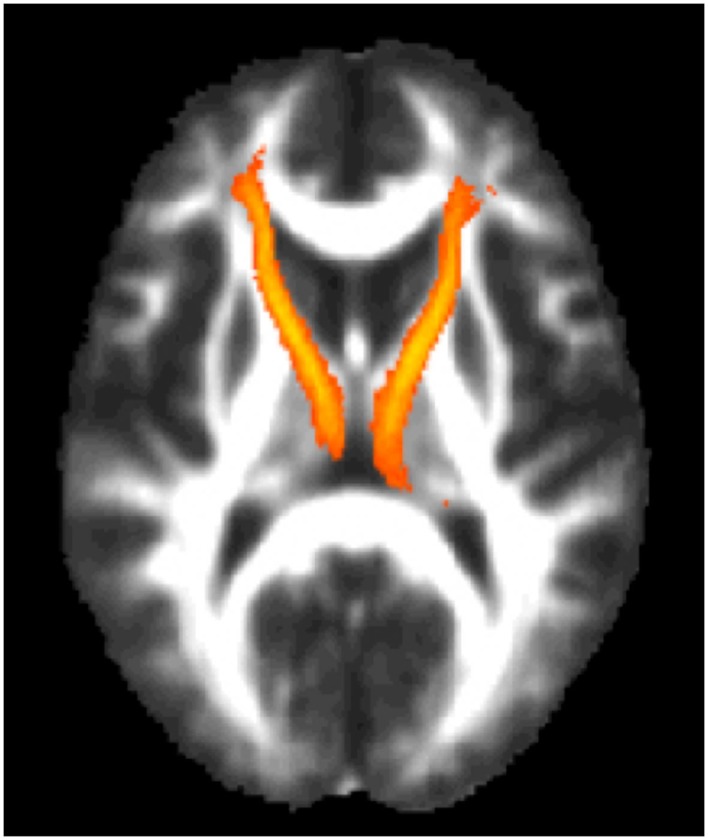
**The region of interest/mask of the bilateral anterior thalamic radiations shown in yellow-orange superimposed on the group mean fractional anisotropy image for the entire cohort**. The region of interest/mask was thresholded to a 75% probability of being within the actual anterior thalamic radiations.

Given the study hypothesis, regions of interests (ROIs) were created for the left and right ATR utilizing the JHU white matter tractography atlas (61, 62). The left and right ATR ROIs were thresholded to minimum of 25 and maximum of 100 to obtain 75% probability of the ROI being within the ATR bundle (See Figure 2). After thresholding, the left and right ATR ROIs were utilized to extract mean FA values for each participant. Next, the left and right ATR were combined and used as a white matter mask and voxel-wise group comparisons were performed via permutation testing in randomize, within FSL. The Threshold-Free Cluster Enhancement option in Randomize was used to eliminate the need to estimate a cluster-forming threshold and 5000 permutations were run for each group comparison and for each regression analysis. Test-statistical maps were produced for group and regression analyses and voxels with a *p* ≤ 0.01 were considered significant. The TBSS_fill command was used within FSL to improve visualization of voxels that were significant.

### Statistics

Statistical analyses were carried out with SPSS software (version 19) for Macintosh. Independent Student’s *t*-tests and Chi-square tests were performed for demographic and clinical data. Left and right thalamic volumes extracted from FreeSurfer were evaluated first by Student’s *t*-tests and then further via Univariate analyses. Thalamic volumes were adjusted by total segmented brain volume (TSV) (volume/TSV × 1000) prior to any analyses. To determine whether or not a laterality effect was present within the thalamus an asymmetry index was calculated for the thalamus using the following formula: (right volume – left volume)/[(right volume + left volume)/2]. Pearson’s correlations were performed between thalamic volumes and mean FA values and clinical variables including suicide and impulsivity measures. Voxel-wise regressions were also performed via permutation testing for clinical variables and total ATR FA data. Cohen’s *d* was calculated for volumetric and mean FA data to determine effect sizes. Because we had specific *a priori* hypotheses regarding thalamic volumes and FA within the thalamic radiations, corrections for multiple comparisons were not performed.

## Results

### Demographic and clinical measures

#### Healthy controls compared to Veterans with TBI and suicide behaviors

There were no significant between-group differences in age, years of education, vocabulary measures, or social status as assessed by the Hollingshead (39); however, a significant difference was found between groups for GAF scores (HC: 80.57 ± 6.97; TBI + SB: 53.42 ± 6.19, *p* < 0.001) (See Table 1). Depression severity, as measured by the HAM-D, was higher in the TBI + SB group relative to controls (HC: 2.73 ± 3.13; TBI + SB: 15.00 ± 7.68, *p* < 0.001) as was severity of anxiety symptoms, as evaluated by the HAM-A (HC: 3.73 ± 5.16; TBI + SB: 14.95 ± 8.13, *p* < 0.001). Increased impulsivity personality traits, as assessed by the BIS total score were found in the TBI + SB group, relative to controls (HC: 67.40 ± 9.19; TBI + SB: 76.11 ± 10.83, *p* = 0.02) a finding that seemed to be driven by the BIS attention subscale score (HC: 18.87 ± 2.61; TBI + SB: 22.00 ± 3.87, *p* = 0.01) as other subscale scores failed to reach significance.

#### Healthy controls and Veterans with TBI and no suicide behaviors compared to Veterans with TBI and suicide behaviors

There were no significant between-group differences in age or vocabulary measures; however, a significant difference was found between all three groups for years of education (*p* = 0.01), social status (*p* = 0.03), and GAF scores (*p* < 0.001). Depression symptom scores were higher in the TBI + SB group relative to the combined HC and TBI-SB group (*p* < 0.01); however, no significant difference was found between groups on HAM-A scores. A significant increase in BIS total score was found in the TBI + SB group relative to the combined group (*p* = 0.02). Subscale BIS scores assessing impulsivity traits related to attention and planning were also significantly increased in the TBI + SB group relative to the combined group (*p* < 0.01 and *p* = 0.03, respectively); however, the subscale score assessing motor impulsivity was not significantly different between groups.

#### Veterans with TBI and no suicide behaviors compared to Veterans with TBI and suicide behaviors

There were no significant between-group differences in age, social status, or vocabulary measures; however, a significant difference was found between groups for years of education (TBI-SB: 14.35 ± 1.99, TBI + SB: 13.53 ± 0.90, *p* = 0.03) and GAF scores (TBI-SB: 67.08 ± 13.83, TBI + SB: 53.42 ± 6.19, *p* < 0.001). Depression severity was higher in the TBI + SB group relative to the TBI-SB group (TBI-SB: 9.78 ± 7.74, TBI + SB: 15.00 ± 7.68, *p* < 0.02); however, no significant difference was found between groups on HAM-A scores.

No significant difference in the overall number of TBI events was found between groups. Furthermore, there were no statistically significant differences between groups in the number of subjects diagnosed with lifetime PTSD or MDD (TBI-SB: PTSD, *n* = 23 (57.5%); MDD, *n* = 23 (57.5%); TBI + SB: PTSD, *n* = 14 (73.7%); MDD, *n* = 14 (73.7%), χ^2^ = 0.26 and 0.26 respectively), or both PTSD and MDD (TBI-SB: *n* = 16 (40.0%); TBI + SB: *n* = 12 (63.2%), χ^2^ = 0.10). In the TBI-SB group, 10 subjects had neither a PTSD nor MDD diagnosis, 7 had PTSD only, 7 had MDD only, and 16 subjects had comorbid PTSD and MDD diagnoses. In the TBI+SB group, 3 subjects had neither a PTSD nor MDD diagnosis, 2 had PTSD only, 2 with MDD only, while 12 subjects had comorbid PTSD and MDD. In the TBI + SB only group, the number of suicide behaviors was 2.05 ± 1.61, number of suicide attempts was 0.95 ± 1.03, and intensity of ideation was 17.00 ± 5.26.

Of the 19 TBI + SB subjects, 13 were taking an antidepressant (68.4%), 4 were on a mood stabilizer (21.1%), 7 were taking a benzodiazepine (36.8%), 3 were on an atypical antipsychotic (15.8%), 12 were taking a hypnotic/non-benzodiazepine (63.2%), and 2 participants were prescribed a stimulant (10.5%). Out of 40 TBI-SB subjects, 14 were taking an antidepressant (35.0%), 4 were on a mood stabilizer (10.0%), 2 were taking a benzodiazepine (5.0%), 4 were on an atypical antipsychotic (10.0%), 12 were taking a hypnotic/non-benzodiazepine (30.0%), and none were prescribed a stimulant. Chi-squared analyses indicated the TBI + SB group had higher frequency of antidepressants (χ^2^ = 0.02), benzodiazepines (χ^2^ = 0.001), hypnotics/non-benzodiazepines (χ^2^ = 0.02), and stimulants (χ^2^ = 0.04) use compared to the TB-SB group.

#### Impact of symptoms of depression and anxiety

Separate general linear model analyses were conducted to examine the impact of depression and anxiety, separately, using left and right adjusted thalamic volumes as the dependent variables, the appropriate groups as a fixed factor, and HAM-A and HAM-D as covariates. Anxiety scores did not significantly impact our original findings between groups (HC vs. TBI + SB: left thalamus *p* = 0.006, right thalamus, *p* = 0.001; TBI-SB vs. TBI + SB: left thalamus *p* = 0.06, right thalamus, *p* = 0.02; HC & TBI-SB vs. TBI + SB: left thalamus *p* = 0.02, right thalamus, *p* = 0.01). Depression scores reduced between group significance in the left thalamus between HC and TBI +SB to a trend level (*p* = 0.07), however, the remainder of the findings still maintained significance (HC vs. TBI + SB: right thalamus, *p* = 0.02; TBI-SB vs. TBI + SB: left thalamus *p* = 0.04, right thalamus, *p* = 0.03; HC & TBI-SB vs. TBI + SB: left thalamus *p* = 0.02, right thalamus, *p* = 0.01).

### Thalamic volumes

Thalamic volumes, adjusted for whole brain volume, for TBI + SB was compared to HC, and both right [*t*(1, 32) = −2.10, *p* = 0.04], and left [*t*(1, 32) = −2.78, *p* = 0.009] volumes were found to be larger in the TBI + SB group. Left and right thalamic volumes were also found to be significantly larger in the TBI + SB group compared to the HC plus the TBI-SB group [*t*(1, 72) = −1.97, *p* = 0.05 and *t*(1, 72) = −2.34, *p* = 0.02, respectively]. When comparing TBI + SB to TBI-SB, again those with a lifetime history of SB had increased volumes in the right thalamus [*t*(1, 57) = −2.34, *p* = 0.02] and demonstrated a trend in the left thalamus [*t*(1, 57) = −1.93, *p* = 0.06] (See Table 2). No significant between group differences were found between HC and the combined TBI-SB and TBI + SB group in left (*p* = 0.53) or right (*p* = 0.62) adjusted thalamic volumes. No significant differences in the asymmetry index for the thalamus was found between HC versus TBI + SB, HC versus TBI-SB, TBI-SB versus TBI + SB, or HC versus TBI-SB and TBI + SB.

**Table 2:** **Between-group differences comparing anterior thalamic radiations fractional anisotropy and thalamic volumes (cm^3^) adjusted for brain segmented volume**.

	HC (*n* = 15)		TBI-SB (*n* = 40)		TBI + SB (*n* = 19)		HC versus TBI + SB		TBI-SB versus TBI + SB		HC and TBI-SB versus TBI + SB	
	Mean	SD	Mean	SD	Mean	SD	*p*	*d	*p*	*d*	*p*	*d*
**VOLUMES**												
Left thalamus	6.72	0.41	6.85	0.47	7.08	0.33	0.01	1.01	0.06	0.54	0.02	0.65
Right thalamus	7.02	0.39	7.03	0.46	7.32	0.43	0.04	0.75	0.02	0.65	0.01	0.67
**FRACTIONAL ANISOTROPY**												
Left anterior thalamic radiation	0.40	0.02	0.39	0.02	0.41	0.02	0.66	0.19	0.07	0.56	0.12	0.44
Right anterior thalamic radiation	0.39	0.02	0.39	0.02	0.40	0.02	0.63	0.21	0.11	0.51	0.17	0.41
Total anterior thalamic radiation	0.40	0.02	0.39	0.02	0.40	0.02	0.63	0.20	0.07	0.52	0.12	0.42

*Effect sizes calculated using Cohen’s d.

### Diffusion tensor imaging

Mean right, left, and total ATR FA values were extracted from each participant and group comparisons were performed via Student’s *t*-tests. FA values were found to be higher in the TBI + SB group compared to the TBI-SB group on a trend level for left ATR [*t*(1, 57) = −1.88, *p* = 0.07] and total ATR [*t*(1, 57) = −1.83, *p* = 0.07]. No significant between group differences were found between HC and the combined TBI-SB and TBI + SB group for left (*p* = 0.12), right (*p* = 0.45), or total (*p* = 0.56) ATR FA values. No statistically significant differences were found between TBI + SB and either HC or the combined HC and TBI-SB group (See Table 2).

**Figure 3:**
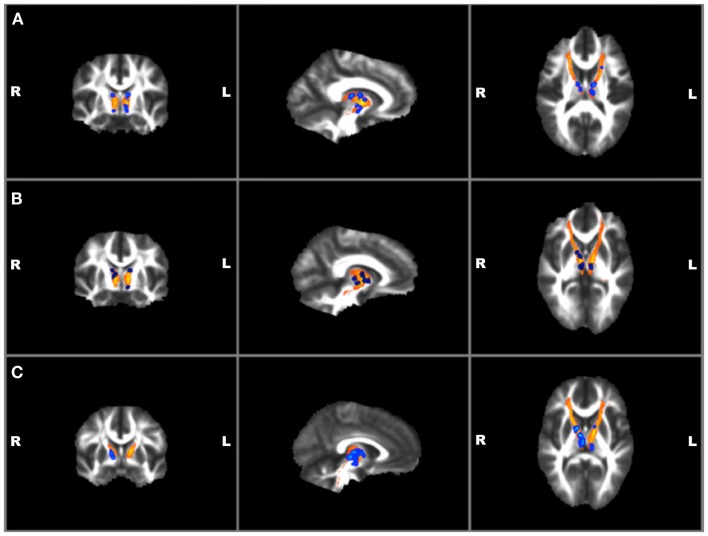
Voxel-wise group analyses of anterior thalamic radiations with regions of significantly (*p* ≤ 0.01)^*^ increased fractional anisotropy shown in blue superimposed on the yellow-orange total (left and right) anterior thalamic radiations mask for (A) individuals with TBI + SB compared to HC; (B) individuals with TBI + SB and the combined group of TBI-SB and HC; (C) individuals with TBI + SB and TBI-SB. ^*^Significant blue regions were filled in by TBSS_fill to improve visualization.

Next, the left and right ATR were combined and used as a white matter mask and voxel-wise group comparisons were performed using permutation testing within FSL. Individuals with TBI + SB had increased FA in bilateral ATR when compared to the combined HC and TBI-SB group and separately with the HC and TBI-SB only groups (*p* ≤ 0.01) (See Figure 3). Additionally, a voxel-wise group analysis comparing HC and the merged all TBI found no significant between-group differences. These findings suggest that mild TBI does not appear to impact the major findings of our study.

### Associations of thalamic volumes and fractional anisotropy in the anterior thalamic radiations

Correlations with clinical and TBI measures were performed on bilateral thalamic adjusted volumes for the TBI + SB group. In the TBI + SB group, the right hemisphere thalamus was significantly correlated with HAM-A score (*r* = −0.49, *p* = 0.03). Adjusted thalamic volumes did not significantly correlate with any TBI measures. Furthermore, in the TBI + SB group, no measure of suicide or impulsivity was found to significantly correlate with adjusted thalamic volumes.

**Figure 4:**
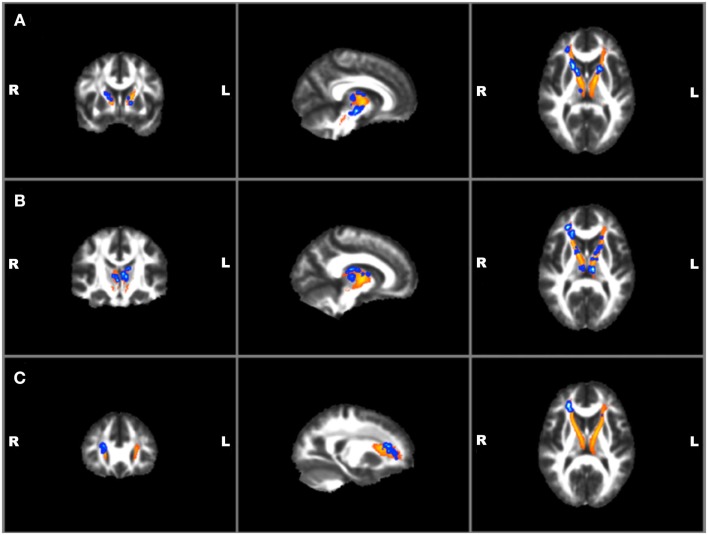
Voxel-wise anterior thalamic regressions in TBI + SB group showing significant (*p* ≤ 0.01)^*^ positive regressions in blue superimposed on the yellow-orange total thalamic radiations mask for (A) Barratt Impulsiveness Scale-Total Score; (B) Barratt Impulsiveness Scale-Planning Score; (C) Hamilton Anxiety scale. ^*^Significant blue regions were filled in by TBSS_fill to improve visualization.

Correlations with clinical and TBI variables and mean FA values for the left and right ATR found a significant positive correlation between BIS total and RT ATR [*r*(19) = 0.47, *p* = 0.04]. No other suicide, TBI, or other clinical measures were found to correlate with mean FA values. Voxel-wise regressions were also performed via permutation testing for clinical variables and total ATR FA data. Significant positive regressions were found for bilateral ATR and HAM-A, BIS total, and BIS planning (*p* ≤ 0.01) and trends were found for bilateral ATR and HAM-D and BIS attention (*p* ≤ 0.05) (See Figure 4). Suicide measures were not found to be significantly associated with ATR. No significant correlation was found between measures of ATR FA and adjusted thalamic volumes.

## Discussion

To our knowledge, this is the first report of enlarged thalamic volumes in a group of male Veterans with TBI + SB as compared to a gender, age, and diagnostically matched TBI-SB cohort and to a HC population. In addition, our study found that the TBI + SB group had greater FA measures in bilateral thalamic radiations compared to both TBI-SB and HC. In the TBI + SB group, right thalamic volumes negatively correlated with HAM-A scores. Total mean FA values for the right ATR positively correlated with BIS total scores. On voxel-wise regression analysis significant positive regressions were found for bilateral ATR and HAM-A, BIS total, and BIS planning and trends were found for bilateral ATR and HAM-D and BIS attention.

The findings of enlarged thalamic volumes in our TBI + SB group are consistent with a post-mortem study of a mixed psychiatric patient population that also reported enlarged thalamic volumes in those individuals who died by suicide (22). Interestingly, the thalamus has one of the highest levels of SERT in the human brain (63) and the 5-HTTLPR genetic variant of SERT has been identified as a major factor influencing emotional behavior and even brain anatomy (64). Individuals with the short SERT allele had 11% larger total thalamic volume than controls (22). Young and colleagues also reported that individuals with major depression had 12% larger total thalamic volumes and individuals who had completed suicide demonstrated an 8% increase in total thalamic volume compared to controls (22). The SERT regulates serotonin levels in the brain by transporting serotonin from the extracellular space into the neuron, thus the altered SERT expression in the short allele variation (23, 24) can affect serotonin availability.

The thalamus has connections with frontal and limbic structures (65, 66). The ALIC is the major efferent tract from the thalamus and carries two major fiber systems, including the ATR and the frontopontine tract (67). The ATR consists of fibers between the anterior thalamic nuclei and both the frontal cortex and anterior cingulate cortices. The anterior thalamic nuclei process afferent information from limbic structures (32). Thus, the thalamus is an important relay station that mediates information flow between the limbic system and the cerebral cortex (65), and thereby participates in the neural processing of cognition (executive function) (32, 68), and mood (69–72). In support of the role of the thalamus in emotion regulation and suicide, studies have reported structural and metabolic abnormalities in the thalamus associated with depressive symptomatology (73–75) and suicide (22, 30).

The short SERT allele has been linked to limbic hyperactivity (27, 76), elevated levels of anxiety and depressive symptoms (27, 77), and an increased incidence of major depression and SB (25–27). For example, the short SERT allele has been linked to amygdala hyperactivity in HC, suggesting that the short allele does not predict mood *per se*, but instead may represent a susceptibility factor for affective disorders (76). Furthermore, in a meta-analysis, Lin and colleagues (26) found no association between 5-HTTLPR polymorphism and suicide; however, among individuals with the same psychiatric diagnosis, those carrying the short allele were significantly more likely to attempt suicide. The short allele was also associated with violent suicide, but not non-violent suicide (26).

In a multimodal imaging study, Pezawas and colleagues (27) reported reduced ACC and amygdala volumes in short SERT carriers compared to individuals with the long SERT genotype. Furthermore the authors performed a functional connectivity analysis with BOLD fMRI data utilizing ACC and amygdala as ROI and noted that the short SERT allele genotype was associated with a greater functional uncoupling between these two regions compared to long SERT carriers. In addition, the functional connectivity between the amygdala and ACC was highly correlated with measures of trait anxiety (27). In contrast, Heinz and colleagues (78) reported an increase in functional coupling between amygdala and medial PFC regions in short versus long SERT carriers suggesting that the short SERT allele can have differential effects on front-limbic circuits. It is unfortunate that neither of the two studies (27, 78) that examined functional connectivity between frontal and limbic regions included the thalamus, given the recent study findings and post-mortem finds of enlarged thalamic nuclei in those with suicide behaviors. However, given that the short SERT genotype has been shown to influence thalamic size (22, 64) one can postulate that changes in fronto-limbic functional coupling in short SERT allele carriers could be related to the thalamic modulation of limbic input into frontal lobe structures.

In the current study we found the TBI + SB group had greater FA measures in bilateral thalamic radiations compared to both TBI-SB and HC. In contrast to our study, Jia et al. (70) reported on a cohort of individuals with MDD, 16 of which were suicide attempters and 36 were not, and found suicide attempters had significantly lower FA in the white matter of the left ALIC as compared to HC and participants with MDD without a history of suicide attempts. In addition, the authors did not find evidence of altered FA (voxel-based or ROI) in bilateral hippocampus or thalamus or an association between HAM-D scores and FA (70). The differences in findings from the current study may be related to the study population (all male Veteran population versus a mixed gender civilian population), in the acquisition parameters used for the diffusion imaging protocol, and data analyses methods (whole brain voxel-wise analysis versus voxel-wise analysis of the ATR region only). Furthermore, in the current study we examined the ATR, which contains white matter fiber projections from the thalamus, through the ALIC and into the frontal cortex. Had we limited our study to the examination of the ALIC, it is possible we would have produced similar findings as Jia and colleagues. Overall, morphometric MRI studies of suicide have yielded findings of larger frontal white matter volumes (17) and high rates of white matter hyperintensities in individuals with SB (13–16, 79). These results indicate that white matter pathology may play a critical role in risk for suicide.

In the TBI + SB group, smaller thalamic volumes were associated with higher HAM-A scores, while DTI analysis found that increased bilateral FA in the ATR were associated with higher HAM-A scores. This apparent disconnect between HAM-A scores and thalamic volumes and ATR FA may be explained by the inclusion of participants with PTSD. Individuals with PTSD often exhibit high levels of anxiety, and PTSD symptoms have been associated with smaller thalamic volumes (80). Functional imaging studies have also reported reduced activation and decreased regional cerebral blood flow in the thalamus in individuals with PTSD (81–84). In addition, a recent study of functional connectivity patterns of the thalamus in individuals with PTSD found two different functional connectivity patterns between the thalamus and the frontal lobes. The authors reported that there was an increased connectivity between the thalamus and inferior and middle frontal gyri and decreased functional connectivity from the thalamus to the medial frontal gyrus and the rostral ACC (85) in individuals with PTSD relative to HC. These findings suggest that different functional fronto-thalamic circuits may be preferentially affected in PTSD. Alternatively, the differential association between HAM-A scores, thalamic volumes, and ATR FA may be due to the inclusion of individuals with TBI, as brain injury, especially DAI, seems to exert the greatest effect on structures in and around the junction between white and gray matter and most often occurs in the frontal and temporal areas, internal capsule, thalamus, and other deep gray matter structures.

In our TBI + SB group, there was a positive association between self-reported levels of impulsivity and FA in bilateral ATR regions. However, we did not find an association between suicidality and either thalamic volume or ATR FA. This could be due to the fact that the suicide measures utilized in this study may not have had enough range (number of lifetime SB) or were not the appropriate measure to detect trait (most severe intensity of ideation) behaviors. However, several other DT-MRI studies have proven effective in observing the relationship between behavioral traits and structural connectivity, particularly impulsivity and suicidality, within the brain. For example, in a study of patients with attention-deficit/hyperactivity disorder, DTI measures of frontal FA were reported to correlate with impulsivity (86). In addition, in a study of Veterans with TBI, the right cingulum FA positively correlated with current suicidal ideation and measures of impulsivity (29). Using the Stroop Task as a measure of inhibition in methamphetamine abusers, Salo and colleagues (87) reported FA values within the genu correlated significantly with measures of cognitive control. In cocaine-dependent subjects, Moeller and colleagues also reported a significant negative correlation between FA in the anterior CC and measures of impulsivity as well as a positive correlation between FA and measures of discriminability (88). Furthermore, in a recent study, Matsuo and colleagues examined the relationship between CC areas, impulsivity, and suicidality in participants with BD (18). The suicidal BD participants had a significant inverse correlation between anterior genu area and the BIS total, motor, and non-planning scores indicating reduced frontal volume in this regions was associated with increased impulsivity (18). Taken together these findings suggest that behavioral measures of impulsivity and suicidality are associated with frontal white matter integrity and that application of DT-MRI to detect white matter associated behavioral changes in individuals with impulsivity and suicide is informative.

There are a number of factors that must be considered when interpreting these study findings. First, the classification of TBI subjects is an area of ongoing debate and the application of different classification criteria can impact study results. Our categorization was based on subject self-report using a structured interview (OSU-TBI) (35), a method that is less sensitive than direct medical documentation. However, given that our study focused on returned Veterans and not active duty personnel, this assessment was feasible and provided an objective quantifiable method for examining Veterans with TBI. Further, our TBI sample was well characterized with current and lifetime history of psychiatric symptoms documented. In addition, our TBI groups were matched on TBI indices, lifetime PTSD, and MDD diagnosis. However, the presence of co-morbid TBI and both MDD and PTSD in our Veteran TBI groups precludes causal inferences regarding the observed findings for thalamic volumes and FA. It is also unknown what impact current and past history of medication may play in our study findings. For example, Ivanov and colleagues (89) reported that ADHD patients receiving stimulant medication demonstrated larger posterior dorsal, ventral, and anterior dorsal thalamic surfaces bilaterally compared to untreated patients. Gilbert (90) and colleagues reported both thalamic volume and symptom severity decreased significantly following 12 weeks of paroxetine treatment in individuals with obsessive-compulsive disorder. Treatment with atypical antipsychotics has also been associated with enlargement of thalamic volumes (91, 92). In a study of individuals with BD, those treated with lithium demonstrated a normalization of thalamic volumes as compared to individuals who did not receive lithium (93).

Thus far, reports on the effects of medication on DTI measures appear inconsistent, although some studies suggest that FA measures may be impacted by medication. For example, white matter FA has been found to be inversely related with mood stabilizer load in individuals with BD (94), antipsychotic dose positively correlated with FA in schizophrenic subjects (95) and antidepressant treatment has been related to significant increases in FA in subjects with obsessive-compulsive disorder (96). However, other studies have found no relationship between psychotropic medications, including antidepressants, mood stabilizers, antipsychotics, and benzodiazepines, and FA in psychiatric disorders (97–101). Additional limitations that should be considered when interpreting the findings of this study include the modest sample size, inclusion of an all male population, and the cross-sectional nature of the study.

In summary, this is one of the first studies to evaluate thalamic structural and microstructural brain changes as measured by FA using DTI and morphometry using MRI in a cohort of Veterans with TBI + SB compared to Veterans with TBI-SB and HC. The present results suggest that Veterans with TBI + SB had enlarged thalamic volumes and increased FA in bilateral ATR as compared to both TBI-SB and HC. Furthermore, the increased FA in bilateral ATR regions was positively associated with self-reported measures of impulsivity. These findings are consistent with previous evidence indicating that suicide may be associated with behavioral disinhibition and frontal-thalamic-limbic dysfunction and suggest a neurobiologic mechanism that may increase vulnerability to suicide. One potential application of these findings may be identifying individuals at-risk for suicide. Further evaluation of key fronto-thalamic-limbic circuits via multimodal imaging combined with candidate genes for SB such as the SERT gene is warranted to ascertain whether or not individuals can be identified as “at-risk” for future suicide. In addition, examining the functional connectivity of the prefrontal-thalamic-limbic circuitry utilizing both resting state and task-based fMRI data in this population would also have the potential to expand current knowledge into the neurobiological underpinnings that may increase risk for suicide. Finally, the thalamus is a region of high susceptibility to diffuse axonal injury in TBI and therefore further study in a high risk Veteran population is needed.

## Conflict of Interest Statement

The authors declare that the research was conducted in the absence of any commercial or financial relationships that could be construed as a potential conflict of interest.

## Footnotes

^1^http://surfer.nmr.mgh.harvard.edu/

^2^http://www.fmrib.ox.ac.uk/fsl
